# Psychological variables of crossFit participants: an up-to-date systematic review

**DOI:** 10.3389/fspor.2026.1870064

**Published:** 2026-06-17

**Authors:** Henrique Silva de Oliveira, Fábio Hech Dominski, Rubia Truppel, Anderson D’Oliveira, Alexandro Andrade

**Affiliations:** Laboratory of Sport and Exercise Psychology, Human Movement Sciences Graduate Program, College of Health and Sport Science of the Santa Catarina State University (UDESC), Florianópolis, Brazil

**Keywords:** exercise dependence, functional fitness, mental health, motivation, psychological variables, sport and exercise psychology

## Abstract

**Introduction:**

CrossFit® presents a relevant context for investigating psychological variables due to its characteristics, including high-intensity training, a competitive environment, strong social interaction, and a focus on performance and overcoming physical and mental limits. This systematic review aimed to broaden understanding of the psychological variables of CrossFit participants by identifying findings, methodologies, and existing research gaps.

**Methods:**

This systematic review was conducted in accordance with the PRISMA guidelines and registered in PROSPERO (CRD420251120994). Searches were performed in SCOPUS, PubMed, SPORTDiscus, Web of Science, EMBASE, Cochrane Library, and Google Scholar. The methodological quality of the included cross-sectional studies was assessed using the Joanna Briggs Institute (JBI) checklist.

**Results:**

Thirteen studies met the inclusion criteria, most of which employed a cross-sectional design. Exercise dependence was the most frequently investigated variable, with prevalence rates ranging from 14.4% to 25.2%. Intrinsic and self-determined motivation predominated among participants and were strongly associated with passion for the sport. Evidence also suggested positive effects on well-being and emotional self-efficacy. Conversely, competitive settings were associated with increased anxiety levels and negative mood changes. Preference and tolerance for high-intensity exercise were positively related to social support.

**Conclusion:**

The findings indicate that CrossFit participation is associated with high levels of intrinsic motivation, passion for the sport, and emotional self-efficacy, which may contribute to psychological well-being. However, the occurrence of exercise dependence, mood fluctuations, and elevated anxiety in competitive contexts highlights the importance of professional monitoring and appropriate training load management. Further research, particularly longitudinal and experimental studies, is needed to strengthen the evidence regarding the psychological outcomes associated with CrossFit participation.

**Systematic Review Registration:**

https://www.crd.york.ac.uk/PROSPERO/view/CRD420251120994, identifier CRD420251120994.

## 1 Introduction

Regular exercise is widely recognized for its positive effects on physical and mental health. Studies indicate improvements in self-esteem (1) a reduction in anxiety and depression (2, 3), increased subjective well-being (4), and the promotion of a higher quality of life (5). In the field of Sport and Exercise Psychology, different psychological variables, such as motivation (6, 7), self-esteem (8), mood (9), anxiety (10, 11), and mental health (12) have been studied in practitioners of various modalities, in order to aid understanding of the factors that influence adherence and benefit psychological well-being and mental health.

In recent years, interest in Functional Fitness modalities (13) has grown significantly, and CrossFit® stands out as one of the most popular practices in this segment (14). Founded in 2000 in the United States, CrossFit® combines different training variables, including weightlifting, gymnastics, and running, among others, promoting intense, varied, and challenging workouts (15). Its rapid expansion, which by 2024 included more than 15 thousand affiliated boxes in approximately 150 countries (16), reflects not only the search for physical benefits, but also the strong psychosocial appeal, including the sense of community, constant challenges, and overcoming personal barriers (17).

In this scenario, CrossFit presents a relevant context for the investigation of psychological variables, due to its specific characteristics, such as the high intensity of training (15), competitive environment (18), strong social interaction among practitioners through building a community (19), and focus on performance and overcoming physical and mental limits (20). These conditions can both favor the development of positive aspects, such as intrinsic motivation and a sense of self-efficacy, and also pose risks, such as increased stress or competitive anxiety (20).

The aforementioned scenario became even more complex with the arrival of the COVID-19 pandemic, which caused significant changes in the physical exercise routine, including the practice of CrossFit. Social isolation measures, the closure of CrossFit boxes, and mobility restrictions led many practitioners to interrupt their training routines or to adapt them to their homes, which may have negatively impacted important psychological variables, such as motivation, mood, and mental health (21). In addition, social isolation and the uncertainty generated by the pandemic increased levels of anxiety, stress, and the feeling of loneliness, factors that can make it difficult to adhere to physical activities and affect psychological well-being (22). On the other hand, some studies indicate that the adaptation of training to home or outdoor environments, combined with the use of digital platforms and participation in virtual CrossFit communities, played an important role in maintaining the engagement of practitioners and preserving the psychosocial aspects of the modality during this period (23). Thus, understanding the psychological variables related to CrossFit is essential to guide intervention strategies that promote mental well-being and the continuity of physical activity in adverse contexts (21–23).

Given the growth of CrossFit practice and related scientific production, it is necessary to systematize and critically analyze the available evidence. Considering our previous review (20) that synthesized existing evidence on this topic, it is important to assess whether recent studies have examined the effects of CrossFit training on mental health outcomes.

Thus, the objective of the present study is to broaden understanding of the psychological variables of CrossFit participants, to identify the main findings, methodologies employed, existing gaps, and implications for research and interventions in Exercise Psychology, through a systematic review of the literature.

## 2 Material and methods

### 2.1 Registration and guidelines

This systematic review was conducted in accordance with the recommendations of the Preferred Reporting Items for Systematic Reviews and Meta-Analyses (PRISMA) (54) and was registered in the International Prospective Register of Systematic Review (PROSPERO) - CRD420251120994.

### 2.2 Search strategy

Studies were searched in SCOPUS (Elsevier), PubMed (National Library of Medicine and National Institutes of Health), SPORTDiscus via EBSCO, Web of Science (main collection – Thomson Reuters Scientific), EMBASE, Cochrane Library, and Google Scholar for grey literature on 17/03/2026. The descriptors of the CrossFit modality and the Psychological Variables were selected based on previous studies (20), and are detailed in the table. To ensure a comprehensive search, the terms were included in the searches in the title, abstract, and keyword fields of each database and are organized in Table 1.

**Table 1:** Search descriptors used in databases.

Search terms	Descriptors
1. Crossfit	“extreme conditioning program*” OR “crossfit” OR “high-intensity functional training” OR “crosstraining” OR “functional fitness”
2. Psychology	“psychology” OR “sport psychology” OR “exercise psychology”
Combination	#1 AND #2

Source: Prepared by the authors (2026).

The Web of Science database was prioritized in decisions about duplicate articles. The searches were carried out in the Main Collection, in the advanced search area, with the terms related to the thematic item and the stipulated period from 2020 to May 2026, the date on which the searches were carried out. The search strategy restricted studies to the period between 2020 and May 2026 because the present manuscript was designed as a complementary update to the review published by our research group (20), rather than as a full replication of the previous review. In addition, the search strategy was completed with a comprehensive search of the “gray” literature, including publications in non-indexed and peer-reviewed journals. Six studies were added from the manual search.

### 2.3 Eligibility criteria

We only included original articles that investigated psychological variables of CrossFit participants through the following topics: attention, activation, cohesion, cooperation, cognition, concentration, coping, feedback, sense of flow, leadership, motivation, satisfaction, self-determination, sense of community, decision-making, mental health, perfectionism, personality, mental training and visualization (related to participation), abandonment, dependence, aggression, anxiety, burnout, dependence, dropout, mood, body image, perception of competence, self-confidence, self-efficacy, self-esteem, depression, emotions, stress, and reaction time (related to the psychological effects of participation). The eligibility criteria were based on the PECOS strategy: Population, Exposure, Comparison, Outcome, Study Design (25), as described in Table 2.

**Table 2:** Eligibility criteria for inclusion of studies in the systematic review.

	Inclusion	Exclusion
P	Any CrossFit participant	Participants in other types of physical exercise
E	CrossFit, high-intensity functional training (HIFT), functional fitness	Massages, manual therapy, stretching, alternative therapies, weight training, walking or running, high-intensity interval training (HIIT)
C	With healthy or unhealthy individuals, with other physical exercise groups or control group without intervention	
O	Psychological aspects	
S	Cross-sectional, randomized, and non-randomized	Case studies, review, meta-analysis

Source: Prepared by the authors (2026).

### 2.4 Study selection and data extraction

Two reviewers (HSO and RTS) performed the search independently and assessed the eligibility of each article. The discrepancies were resolved by a third researcher (AA). All titles and abstracts included were analyzed, and the full text of the articles that met the inclusion and exclusion criteria predetermined in Table 2 was reviewed. The authors (HSO and RTS) independently extracted data from all included studies.

The following data were extracted: title, authors, journal, year of publication, place of study, purpose of the studies, study design, sample (number of subjects, gender, age, and level), type of intervention, psychological variables investigated, instruments used, and main results of the study. Because our goal focused on a wide range of psychological factors, we divided the discussion section according to the prevalence of the psychological variables studied.

For the selection of articles, Rayyan software, developed by the Qatar Institute of Computer Research, was used (26). The other steps were carried out in Microsoft Excel spreadsheets. References in the included articles were reviewed to identify other potentially relevant studies.

### 2.5 Quality assessment

The critical evaluation checklist of the Joanna Briggs Institute (JBI) was used for cross-sectional studies (27). In this JBI checklist, each question is answered by one of four options: yes (S), no (N), unclear (U), and not applicable (NA). The percentage risk of bias is calculated by the number of responses (S) in the checklist. When the selected answer is (NA), the question is not considered for the calculation. In the final sum, 0%–49% is considered as high risk of bias, 50%–70% as moderate risk, and above 70% as low risk of bias.

Two researchers (HSO and RTS) performed the evaluation independently, and the discrepancies were resolved by a third researcher (AA). The quality of the study was not considered as an inclusion or exclusion criterion.

## 3 Results

**Figure 1:**
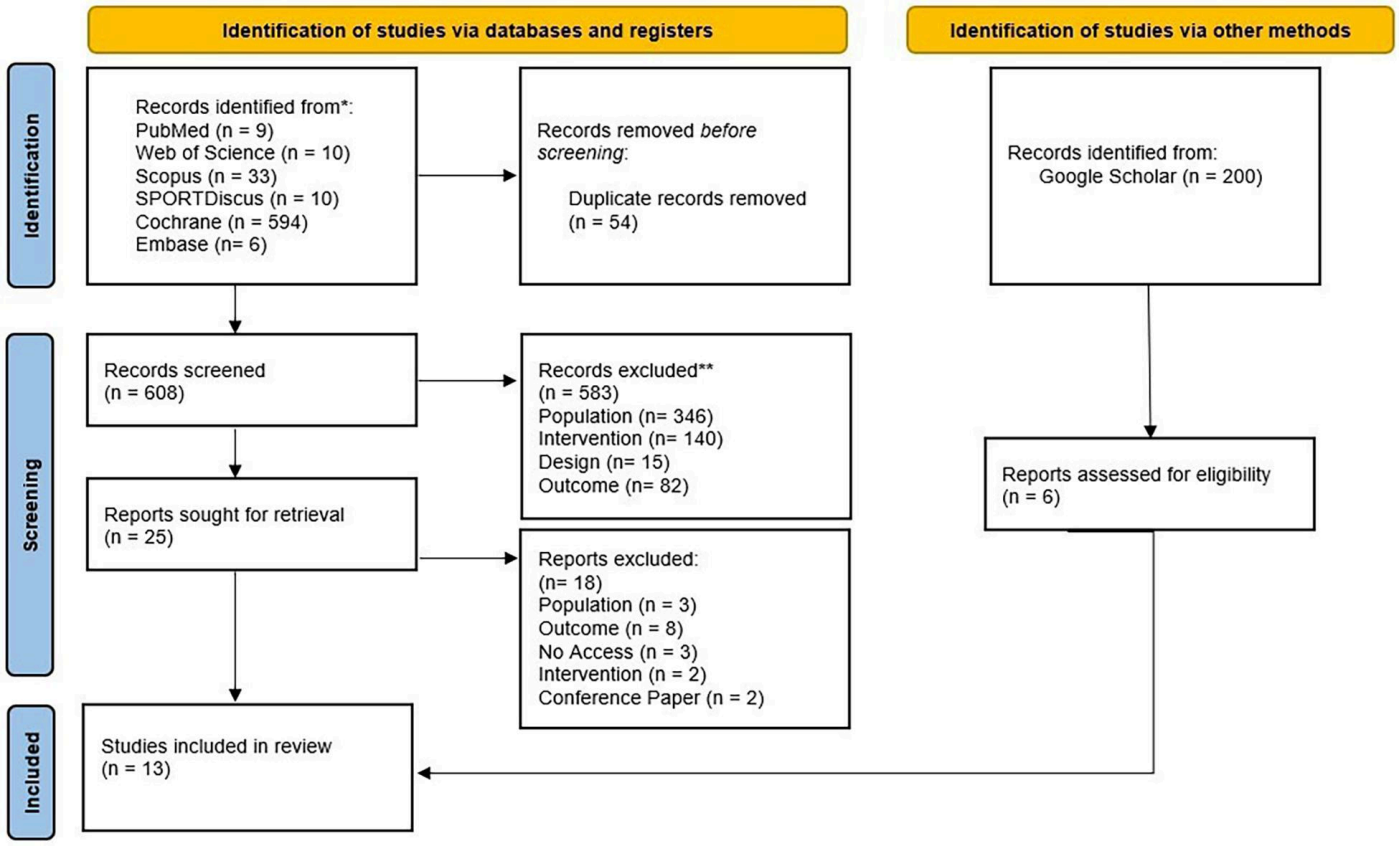
Flow diagram of the included studies.

In the first stage of the database search, 662 articles were found, of which 54 duplicates were excluded and 608 were selected for reading of the title and abstract. In the summary stage, only 25 texts were read in full and, of these, only 7 met the inclusion criteria and were selected for analysis. In addition, six studies were selected from the search in the “gray” literature, adding up to a total of thirteen selected studies, as shown in the PRISMA flowchart in Figure 1.

After selecting the studies and extracting the data, analyses were performed regarding the study design: cross-sectional, experimental, and qualitative. The following data were extracted: title, authors, journal, year of publication, study objective, sample (number of subjects, gender, age, and level), time of practice, weekly frequency, level of experience, study location, psychological variables, instruments, study design, type of intervention, and main results of the study. Because our goal focused on a wide range of psychological factors, we divided the discussion section according to the prevalence of the psychological variables studied. If findings related to a variable appeared in at least two studies, then this was considered a specific topic in the discussion, and the variables in one topic were grouped together.

The following exercise psychology themes were identified: psychological capital (PsyCap), well-being, anxiety, exercise preference and tolerance, personality, depression, sense of community, exercise dependence, adherence, emotional self-efficacy, and motivation.

### 3.1 Research overview

In total, the 13 studies included 3,235 participants of both sexes, made up of 2,762 CrossFit practitioners and 473 participants from other modalities who made up the control group.

The studies used quantitative designs (cross-sectional *n* = 11 and longitudinal *n* = 2). Tables 3–5 presents the characterization of the sample, research design, and location of the studies.

**Table 3:** Study sample, design, and location.

Study	Sample n/gender/age	Experience level	Study design	Study location
(28)	*n* = 85 M = 42.3% F = 57.6% MY = 29.24	Miscellaneous	cross-sectional	Brazil
(29)	*n* = 139 M = 63% F = 37% MY = 43.0	Miscellaneous	cross-sectional	USA
(30)	*n* = 127 M = 33.1% F = 66.9% MY = 35.55	Miscellaneous	cross-sectional	USA
(31)	*n* = 197 M^1^= 50% F^1^= 50% M^2^= 47.2% F^2^= 52.7% M^3^= 31.8% F^3=^ 68.1% MY^1^= 36.56 MY^2^= 43.3 MY^3^= 33.57	Miscellaneous	cross-sectional	USA
(32)	*n* = 263/NR/NR	Miscellaneous	cross-sectional	Brazil and Portugal
(33)	*n* = 159 M = 48.4% F = 51.5% MY = 31.59	Miscellaneous	cross-sectional	Brazil
(34)	*n* = 14 M = 50% F = 50% MY = 25-40	Competitors and Recreationals	Longitudinal	Brazil
(35)	*n* = 241 M = 51.5% F = 48.5% MY = 26.3	Miscellaneous	cross-sectional	Greece
(36)	*n* = 368 M = 42.67% F = 57.3% NR	Miscellaneous	cross-sectional	Brazil
(40)	*n* = 507 M = 62.7% F = 37.3% MY = 38	Miscellaneous	cross-sectional	Hungary
(37)	*n* = 405 M = 55.3% F = 44.7% MY = 30.2	Miscellaneous	Longitudinal	Brazil
(38)	*n* = 202 M = 53% F = 43% MY = 34.30	Miscellaneous	cross-sectional	Italy
(39)	*n* = 325 F = 100% MY 33	Miscellaneous	cross-sectional	USA

Source: Prepared by the authors (2026).

n, number of participants; M, male; F, female; MY, mean age; NR, not reported.

**Table 4:** Main results of studies with psychological variables in crossFit.

References	Psychological variable(s)	Instrument(s)	Main results
(28)	Exercise Dependence	Exercise Dependence Scale Revised (EDS-R)	Women reported higher perceived benefits from CrossFit practice than men. No significant differences in exercise dependence were observed across age groups. However, longer practice duration and higher weekly training frequency were associated with greater perceptions of exercise dependence among CrossFit practitioners.
(29)	Psychological Capital; Wellness	Psychological Capital Questionnaire (PCQ-12); WHO-Five Well-Being Index (WHO-5)	The results showed significant associations between weekly training frequency, psychological capital (PsyCap), and well-being, but not engagement. Membership duration was negatively associated with PsyCap and showed no relationship with well-being or engagement. PsyCap and well-being mediated the relationship between weekly training frequency and engagement, whereas no mediation effects were observed for membership duration.
(30)	Self-efficacy; Enactive mastery; Vicarious experience; Verbal persuasion; Emotional arousal	Multiple Intelligences Self-Efficacy Inventory MISEI-R; sense of community scale (SCS; Warner et al., 2013); Compulsive Exercise Test (CET).	Vicarious experience was the only social–environmental theory (SET) construct associated with self-efficacy for effort and endurance in CrossFit networks, with density, proximity, and network effects also related to effort self-efficacy. Enactive mastery and vicarious experience were associated with self-efficacy for agility, balance, and strength. No SET constructs significantly predicted self-efficacy for coordination.
(31)	Preference and Tolerance; Personality, Depressive Symptoms, Sense of Community	Preference for and Tolerance of the Intensity of Exercise Questionnaire (PRETIE-Q); Ten-Item Personality Inventory (TIPI); Personal Health Questionnaire (PHQ-8); Sense of Community in Sport Scale (SCS)	Local network autocorrelation models (LNAMs) explained 18%–55% of the variance in preferences, with significant network effects observed across all three CrossFit gyms. Transitivity and reciprocity increased the likelihood of social ties, and individuals of the same gender or with longer membership duration were more likely to connect. Lower tolerance scores and depressive symptoms were also associated with network connections across the gyms.
(32)	Motivation	Online questionnaire prepared by the authors	Participants maintained high levels of motivation and training routine even with restrictions
(33)	Exercise Dependence	Exercise Dependence Scale (EDS-R)	The main results revealed that only the frequency of training was associated with ED. the time of practice did not present a significant prediction with the dimensions and the overall score of ND. It was noticed that age was also not shown to be a predictor for the development of ED.
(34)	Humor; Stress	Escala de Humor BRUMS; Recovery-Stress Questionnaire for Athletes	Among competitors, tension decreased between periods 1–2 and 1–3, while anger also declined over time. Vigor decreased significantly between periods 1–2 and 2–3. In non-competitors, anger and vigor increased between periods 1–2, with vigor remaining higher in period 3. No significant intragroup differences were observed for fatigue. The non-competitive group maintained an iceberg profile throughout the study, whereas competitors showed a temporary deterioration in mood profile during period 2, marked by reduced vigor and increased negative mood states, followed by recovery in period 3.
(35)	Exercise Dependence; Orthorexia Nervosa; Perfectionism	The Exercise Dependence Scale-Revised (EDS-R; Downs et al., 2004); ORTO-15 scale, Almost Perfect Scale	The exercise dependence scale, on which crossfitters scored higher (M = 66.06, SD = 19.55) than gym members (M = 61.39, SD = 16.92) at a significant level [t (241) = 1.988, *p* < 0.05, effect size = 0.25]. In addition, 3.8% of gym members and 19.8% of crossfitters were at risk for exercise addiction. Gender was considered a statistically significant predictor of the ORTO-15 score (B = 1.14, SE = 0.58, *p* < 0.05). It was revealed that men were more likely to express orthorexic behaviors compared to women.
(36)	Exercise Dependence; Quality of life	Exercise Addiction Inventory (EAI); WHOQOL-bref	Among CrossFit practitioners, 39.1% participated in another sport. Exercise dependence prevalence was 13.2% overall and 15.2% among those practicing only CrossFit. Dependence showed weak positive correlations with the physical domain and weak negative correlations with the environmental domain of quality of life. Additionally, exercise dependence was significantly associated with the environmental domain.
(Trench; Szabó, 2025)	Exercise addiction; Paixão (OP/HP); Sports motivation	Exercise Addiction Inventory-3 revisado (EAI-3); Passion Scale; Sport Motivation Scale (H-SMS)	Women practicing CrossFit showed higher intrinsic and introjected regulation scores than controls, whereas men scored lower only for introjected regulation. High risk of exercise addiction (H-REA) was more prevalent among CrossFit practitioners (25.2%) compared with mixed-exercise participants (13.4%). Perceived exercise intensity significantly influenced exercise addiction, obsessive passion, and harmonious passion, with CrossFit practitioners reporting greater workout exertion than controls.
(37)	Membership	Data analysis of Box's management system	57% (*n* = 231) stopped monthly payments to the HIFT program/facility after three months, 75.9% (*n* = 291) after six months, 80.5% (*n* = 326) over nine months, and 87.2% (*n* = 353) after 12 months. Individuals’ likelihood of HIFT gym adherence decreased over time. There was no difference in the probability of adherence curves for women versus men. Similarly, there was no difference in the curves of probability of adherence between the age groups.
(38)	Psychosocial Well-Being	Online questionnaire prepared by the authors	95% of respondents indicated a reduction in stress levels, 73.3% reported better sleep quality, and more than 90% noticed improvements in self-esteem and daily energy. In addition, 98.5% of participants formed new social connections through CrossFit ®, with 79.2% recognizing a positive impact on social relationships. No significant gender differences were found in most psychological and social outcomes.
(39)	Eating disorder, exercise dependence, risk of body dissatisfaction.	Eating Disorder Examination Questionnaire (EDE-Q); Exercise Dependence Scale (EDS-21) e questionário elaborado pelos autores	Among CrossFit athletes, 28.9% reported a history of stress fractures and 54.7% reported a past or current eating disorder. Most trained 4–6 days per week. Overall, 37.2% were at risk for eating disorders, and 84.6% showed symptomatic eating disorder behaviors. The competitive obesity (CO) group demonstrated higher eating disorder risk and greater exercise dependence scores than the comparison exercise (CE) group, particularly for lack of control, intention, and reduction of other activities. Many athletes also reported inadequate nutritional intake, avoidance of specific food groups, and reliance on non-specialized dietary guidance from coaches.

Source: Prepared by the authors (2026).

**Table 5:** Critical evaluation of eligible cross-sectional analytical studies.

Study	Q1	Q2	Q3	Q4	Q5	Q6	Q7	Q8	Total %	Risk
(28)	Y	Y	Y	Y	N	N	Y	Y	75	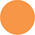
(29)	Y	Y	U	Y	N	N	Y	Y	62	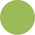
(30)	Y	Y	U	Y	N	N	U	Y	50	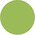
(31)	Y	Y	Y	Y	Y	Y	Y	Y	100	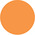
(32)	Y	Y	N	Y	N	N	Y	Y	50	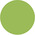
(33)	Y	Y	Y	Y	N	N	Y	Y	75	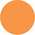
(35)	Y	Y	Y	Y	Y	N	Y	Y	87	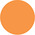
(36)	Y	Y	Y	Y	N	N	Y	Y	75	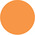
(Trench; Szabó, 2025)	Y	Y	Y	Y	Y	N	Y	Y	87	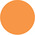
(38)	Y	Y	N	Y	N	N	U	Y	50	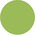
(39)	Y	Y	Y	Y	U	N	Y	Y	75	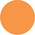

Source: Prepared by the authors (2026).

Q1. Were the criteria for inclusion in the sample clearly defined? Q2. Were the study subjects and the setting described in detail? Q3. Was the exposure measured in a valid and reliable way? Q4. Were objective, standard criteria used for measurement of the condition? Q5. Were confounding factors identified? Q6. Were strategies to deal with confounding factors stated? Q7. Were the outcomes measured in a valid and reliable way? Q8. Was appropriate statistical analysis used?.

Y, Yes; N, No; U, Unclear; 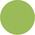, Low risk of bias; 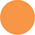, Moderate risk of bias; 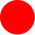, High risk of bias.

### 3.2 Main results

Most studies had a cross-sectional design, with samples composed of young adults, of both sexes, with varying levels of experience in the modality, and one study separated competitors from recreational practitioners. The most recurrent variable analyzed was dependence on exercise (27, 28, 33, 35, 36, 40), but other important variables were also researched, such as motivation (32), mood, and stress (34). Validated instruments such as EDS-R, EAI, BRUMS were used in the studies.

The prevalence of traits of dependence on exercise ranged from 14.4% to 25.2%, with emphasis on associations with sex, perfectionism, and time and frequency of training (27, 28, 33, 35, 36, 40). Regarding motivation, intrinsic and self-determined motivation predominated, with a strong influence of passion for practice (27, 39). The available evidence suggests potential benefits regarding well-being and emotional self-efficacy among CrossFit participants (29, 30), while the competitive environment negatively impacted mood and raised anxiety levels (34). Preference for and tolerance to intense exercise were also associated with perceived social support in the box environment (31).

### 3.3 Quality assessment

Only cross-sectional studies were evaluated, since they represent most of the production found. Thus, two longitudinal studies were not included in the evaluation. Among the eleven studies analyzed, six were classified as low risk of bias, five as moderate risk, and none were classified as high risk of bias.

## 4 Discussion

The current work is an update of a previous systematic review (20) by our research group, which comprehensively and in an in-depth manner synthesized the state of scientific production on the psychological variables of CrossFit practitioners in the period from 2020 to 2026. The update is necessary due to the advancement of research in the area that generates new evidence and more robust investigations and the use of validated instruments to assess variables such as mood, exercise dependence, and well-being. In addition, the post-COVID-19 pandemic context may have influenced the psychological aspects of CrossFit practitioners (32, 41).

Compared to the previous review (20), there was an increase in the number of studies that addressed psychological aspects, as well as diversification in the variables analyzed. The inclusion of new themes, such as emotional self-efficacy, and obsessive and harmonic passion, in addition to the approach to contemporary themes, such as social network analysis, demonstrate advances in the field.

### 4.1 Exercise dependence

The topic dependence on exercise was the most commonly investigated among the studies analyzed. This condition is characterized by compulsive and excessive involvement in physical activities, which can cause damage to physical and psychological health (42). Although it can also be associated with positive aspects such as better physical performance, it often results in damage to mental, social, and physical health, including a drop in quality of life and well-being (36, 41). Traits of perfectionism are consistently associated with exercise dependence, especially in high-demand contexts such as CrossFit and competitive sports (35, 42). High weekly frequency and longer practice time are robust predictors of dependence, with athletes and regular practitioners having a higher risk (28, 33, 43), in addition to female practitioners, with longer practice time demonstrating a higher risk of dependence in standard CrossFit (28). These findings differ from those observed in fighting modalities, such as Kickboxing, Taekwondo, and Muay Thai (44).

### 4.2 Motivation and adherence

Motivation in CrossFit practitioners was predominantly intrinsic, based on autonomy, personal challenge, and sense of competence (27, 39), corroborating the findings of previous literature (20, 45, 46). Passion for the modality, both harmonic and obsessive, also appeared as a relevant motivational factor (27). The authors revealed that obsessive passion (OP) and harmonic passion (HP) were significantly higher among CrossFit practitioners compared to practitioners of other types of exercise, with OP and HP being stronger predictors than exercise dependence itself. This suggests that passion can be confused with addictive behaviors when not analyzed together. Men tend to pursue more performance goals, while women focus on mastery goals (38, 47). Although the time of practice can influence the type of goal (adherence vs. permanence), pleasure and health remain the main motivators for both sexes.

Patterson et al. (31) showed that preference and tolerance for high-intensity exercise are associated with social factors, such as integration into the social environment of the box, and have pleasure and adherence as a response. Constant exposure to intense challenges and the support of colleagues seem to strengthen these psychological variables, favoring permanence in the program. Adherence to High-Intensity Functional Training (HIFT) tends to be low to moderate in the long term, with greater dropout in the first few months. Social, motivational, and self-efficacy factors strongly influence the permanence of practitioners (37), which has not been observed previously (20).

### 4.3 Mood

Previous studies suggest that CrossFit sessions can promote acute reductions in negative mood states, such as fatigue and tension, even under conditions of high intensity and high perceived exertion. However, this apparent positive response should be interpreted with caution, since it may reflect transient effects of exercise, that are not necessarily sustained over time or under different load conditions (20, 48). In addition, the absence of strict control of training variables in some of the studies limits understanding of the threshold at which exercise ceases to have a beneficial effect and starts to contribute to greater psychological exhaustion.

Mood has been predominantly investigated in competitive contexts, in which emotional demands are amplified (34). In these scenarios, it has been observed that athletes tend to present a positive mood profile in the pre-competition period, characterized by high vigor and low levels of negative states. However, this pattern seems to change in the post-competition period, with an increase in negative emotions and a reduction in vigor (2, 34, 49). Despite this, such results are not unanimous and may be influenced by contextual factors, such as competitive level, previous experience, performance expectations, and results obtained, aspects that are not always controlled or consistently reported.

**Figure 2:**
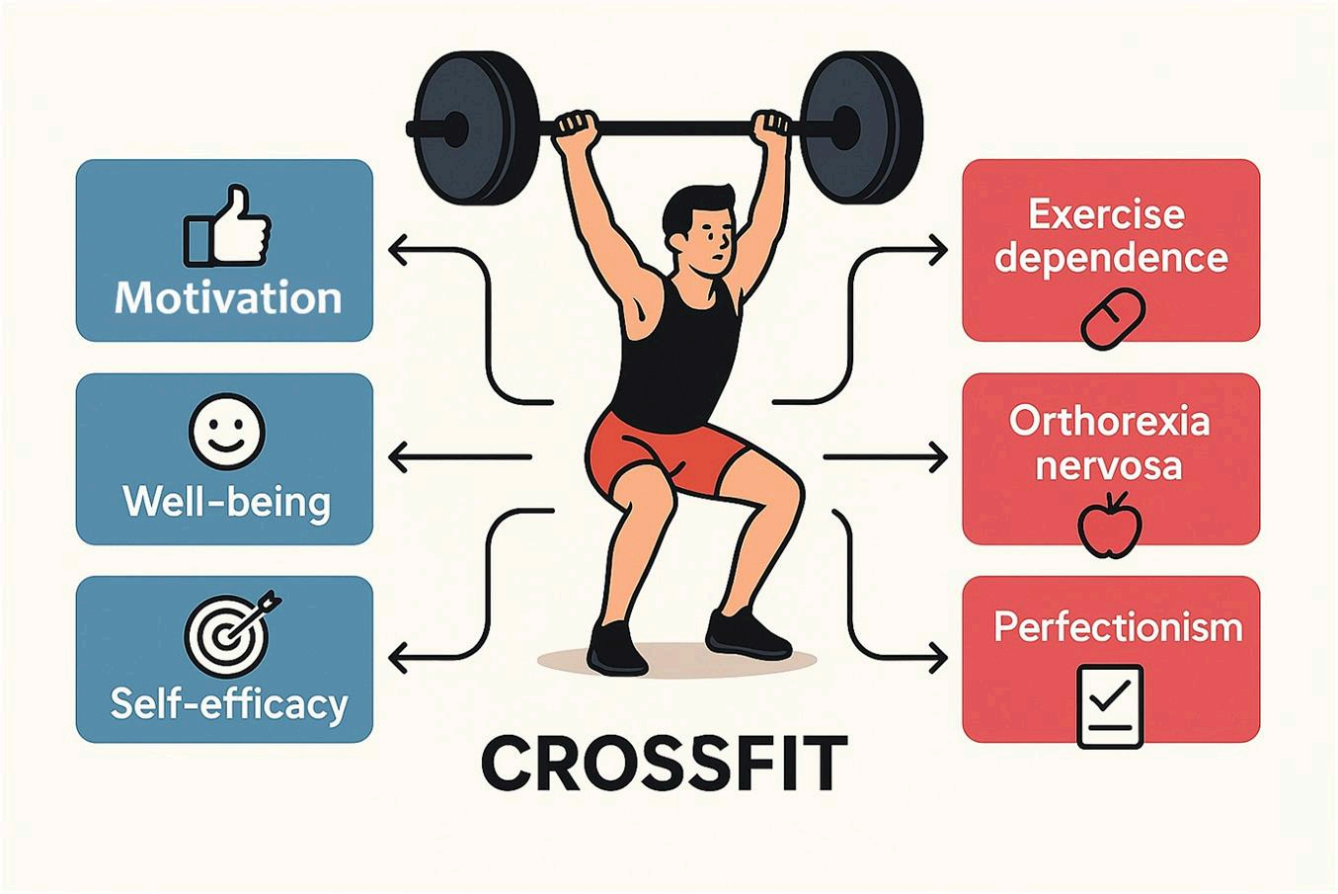
Positive effects and points of attention.

Thus, although CrossFit has the potential to positively modulate mood in certain situations, the available findings still lack greater methodological standardization and control of variables. The competitive context seems to act as a factor of intensification of emotional responses, which can have heterogeneous repercussions on both performance and psychological recovery processes, reinforcing the need for investigations that consider the complexity of these interactions. These findings are summarized in Figure 2.

### 4.4 Key findings of the review

The present review showed that the practice of CrossFit is associated with important psychological outcomes, characterized by duality between mental health benefits and potential behavioral risks.

Regarding the positive impacts, one included study reported that approximately 95% of practitioners experienced reductions in stress levels, often accompanied by improvements in sleep quality and increased daily disposition (46). In addition, intrinsic motivation emerged as one of the main factors associated with adherence, supported by elements such as autonomy, sense of competence, and the pursuit of personal challenges (27, 39). Another included study found that 98.5% of participants reported developing new social connections, which may contribute to greater engagement and tolerance to high-intensity exercise (46).

On the other hand, the findings also indicate points of attention. The prevalence of risk of exercise dependence ranged between 14% and 25%, being associated with factors such as high training frequency and perfectionism traits (27, 28, 33, 35, 40). In addition, competitive environments can favor mood swings, including increased anxiety and decreased vigor in the post-competition period (34). A high dropout rate was also observed over time, reaching approximately 87% after 12 months, suggesting challenges in maintaining adherence in the long term (37).

**Figure 3:**
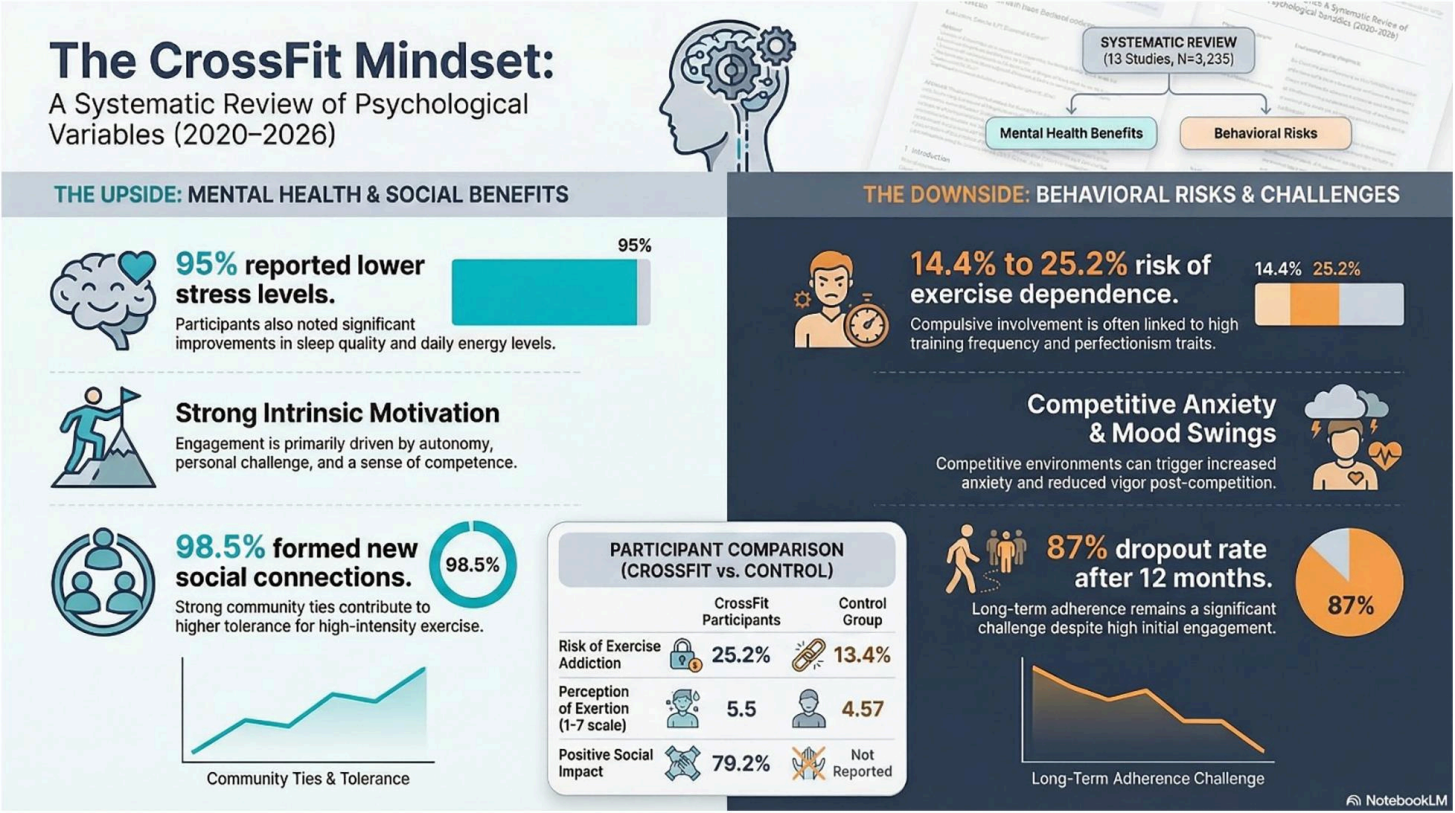
Key study findings.

Compared to traditional exercisers, CrossFit individuals had a higher risk of dependence (25.2% vs. 13.4%) (27), a higher perception of exertion (5.5 vs. 4.57 on a 7-point scale) (27), and a greater positive impact of social connections (79.2%), a variable not reported in the control groups. The main findings presented by the authors are summarized in Figure 3.

### 4.5 Limitations and future studies

Among the main limitations of this review, the fact that the included studies were conducted in only a few countries stands out, restricting the generalization of the results to different sociocultural contexts. In addition, most of the studies had a cross-sectional design, which limits understanding of causal relationships between the variables analyzed. The small number of studies that met the inclusion criteria also represents a limitation, especially considering the diversity of variables investigated, which makes direct comparisons and the construction of more robust conclusions difficult. In this sense, studies with experimental designs, representative samples, and validated instruments are still needed to strengthen the evidence and allow more consistent conclusions about the psychological effects of CrossFit.

### 4.6 Strengths, innovations, and applications

The findings of the current study reinforce the need for multidisciplinary work with sports psychologists and physical education professionals. Thus, regular psychological monitoring in active populations, combined with strict control of the volume and intensity of training loads, is essential to promote performance, prevent injuries, and avoid negative effects such as overtraining and decreased well-being.

Psychological indicators (such as perceived stress, mood, and recovery) directly reflect the response to training and can predict a significant part of the variation in training load, as well as aiding in injury prevention and training adjustment (50). Load control involves combining objective (heart rate, accelerometers, training volume) and subjective (perceived exertion scale - RPE, workout diaries) methods to quantify both external and internal load (51, 52). Integration of psychological and physiological data allows more precise adjustments in training, reducing the risk of injury, chronic fatigue, and decreased performance (51–53). Therefore, integration of regular psychological monitoring with strict control of the volume and intensity of loads is essential for the health, safety, and performance of active populations, and should be adapted to individual needs and specific contexts.

## 5 Conclusion

The objective of the current review was to investigate the psychological variables of CrossFit practitioners, to identify which variables are most commonly studied, through a systematic review. The review allows us to conclude that CrossFit, as a modality of high-intensity exercise, influences a wide range of psychological variables. CrossFit participation appears to be associated with high levels of intrinsic motivation, passion for the sport, and emotional self-efficacy, suggesting potential benefits for the psychological well-being of its practitioners. However, aspects such as exercise dependence, mood swings, and high levels of anxiety in competitive contexts reinforce the need for professional monitoring in relation to adequate load control. Thus, it is essential that coaches are aware of the signs of imbalance, in order to maximize the benefits while minimizing the risks associated with the practice of CrossFit.

## Data Availability

The original contributions presented in the study are included in the article/Supplementary Material, further inquiries can be directed to the corresponding author.
